# Weather-based prediction of *Plasmodium falciparum *malaria in epidemic-prone regions of Ethiopia I. Patterns of lagged weather effects reflect biological mechanisms

**DOI:** 10.1186/1475-2875-3-41

**Published:** 2004-11-12

**Authors:** Hailay D Teklehaimanot, Marc Lipsitch, Awash Teklehaimanot, Joel Schwartz

**Affiliations:** 1 Department of Epidemiology, Harvard School of Public Health, 677 Huntington Avenue, Boston MA 02115, USA; 2 Mailman School of Public Health, Columbia University, New York, NY, USA; 3 Department of Environmental Health, Harvard School of Public Health, 677 Huntington Avenue, Boston, MA 02115, USA

## Abstract

**Background:**

Malaria epidemics due to *Plasmodium falciparum *are reported frequently in the East African highlands with high case fatality rates. There have been formal attempts to predict epidemics by the use of climatic variables that are predictors of transmission potential. However, little consensus has emerged about the relative importance and predictive value of different factors. Understanding the reasons for variation is crucial to determining specific and important indicators for epidemic prediction. The impact of temperature on the duration of a mosquito's life cycle and the sporogonic phase of the parasite could explain the inconsistent findings.

**Methods:**

Daily average number of cases was modeled using a robust Poisson regression with rainfall, minimum temperature and maximum temperatures as explanatory variables in a polynomial distributed lag model in 10 districts of Ethiopia. To improve reliability and generalizability within similar climatic conditions, we grouped the districts into two climatic zones, hot and cold.

**Results:**

In cold districts, rainfall was associated with a delayed increase in malaria cases, while the association in the hot districts occurred at relatively shorter lags. In cold districts, minimum temperature was associated with malaria cases with a delayed effect. In hot districts, the effect of minimum temperature was non-significant at most lags, and much of its contribution was relatively immediate.

**Conclusions:**

The interaction between climatic factors and their biological influence on mosquito and parasite life cycle is a key factor in the association between weather and malaria. These factors should be considered in the development of malaria early warning system.

## Background

Malaria epidemics due to *Plasmodium falciparum *are reported frequently in the East African highlands [1-6]. Immunity to malaria in the populations of these epidemic-prone regions is often incomplete, so that epidemics cause high case fatality rates among all age groups. In 1958, a malaria epidemic covering over 250,000 square kilometers resulted in an estimated three million cases and 150,000 deaths in Ethiopia [2]. Since then, large scale epidemics of malaria have been noted every five to eight years. Thus, there is an urgent need for the development of malaria early warning systems [7-9] to predict where and when malaria epidemics will occur, with adequate lead-time to target scarce resources for prevention activities. Unusual meteorological conditions, such as especially high rainfall or high temperature, are often cited retrospectively as the precipitating factors for epidemics [10,11]. There have also been formal attempts to predict epidemics by the use of local weather and/or global climatic variables that are predictors of vector abundance and, therefore, of transmission potential [12-20]. However, little consensus has emerged about the relative importance and predictive value of different factors [2,6,21-27]. Woube [27] showed that although one epidemic in Ethiopia was associated with higher rainfall, an epidemic in another year was preceded by very little rainfall. Lindsay et al. [23] found a reduction in malaria infection in the Usambara Mountains of Tanzania following 2.4 times more rainfall than normal, while excessive rainfall during the same period was associated with increased malaria in south-western highlands of Uganda [3]. Moreover, Mbogo et al. [24] found variation in the relationship between the mosquito population and rainfall in different districts of Kenya and attributed the variation to environmental heterogeneity. Similarly, Zhou et al. [28] showed that there was high spatial variation in the sensitivity of malaria outpatient numbers to climate fluctuations in East African highlands. Similarly, determination of the amount of lead time between weather factors and malaria cases is necessary to develop prediction models, but results from different studies have revealed a range of lead times [3,4,22,24,29]. Despite the varying results of these studies, there has not been critical examination of the sources of variation in the association and lag structure (the magnitude of association between weather and malaria at a later time) between weather and incidence of malaria.

The inconsistency of findings from different studies may be due in part to the interaction of weather factors affecting vector abundance and survival, and parasite maturation, key determinants of malaria transmission. Deposition of mosquito eggs, and their maturation into larvae and then into adults, requires aquatic breeding sites, and is, therefore, dependent on rainfall [30,31]. The time required for mosquito maturation shortens as temperature increases [30,32]. At 16°C, larval development may take more than 45 days (reducing the number of mosquito generations and putting the larvae at increased risk of predators), compared to only 10 days at 30°C (Table 1). By affecting the duration of the aquatic stage of the mosquito life cycle, temperature determines the timing and abundance of mosquitoes following adequate rainfall.

**Table 1:** The effect of mean temperature on the duration of mosquito's life cycle and sporogonic cycle and its effect on the amount of lead time from the availability of breeding sites to the occurrence of malaria cases.

***Weather factors***	***Stages and duration of mosquito's life cycle and sporogony cycle affected by weather factors***		
**Mean temperature (Rainfall temperature)**	**Availability of breeding sites ----------------→ Malaria**		
	
	Mosquito's life cycle*	**Sporogony†**	**Incubation period in human host**
		
	Larva ----→ Adult(days)	Adult first bite ----→ Infectious bite (days)	
	
16°C	47	111	**(10–16 days)**
17°C	37	56	
18°C	31	28	
20°C	23	19	
22°C	18	7.9	
30°C	10	5.8	
35°C	7.9	4.8	
39°C	6.7	4.8	
40°C	6.5	4.8	

* see references [32, 44]; †see references [35, 45]

Once female adult mosquitoes emerge, they look for a blood meal, and in the process they ingest malaria parasites (gametocytes) with the blood. The feeding frequency of mosquitoes increases with temperature, resulting in increased proportions of infective mosquitoes [33]. The duration of the extrinsic phase of the parasite (sporogony cycle), which is the development of the ookinete, the egg of the parasite, in the midgut of the anopheline mosquito, depends on temperature. The sporogony cycle on average lasts about 10 days, but shortens as temperature increases [34,35], becoming as short as five days when the temperature exceeds 30°C (Table 1).

These data on the timing of the mosquito life cycle suggest that malaria cases should follow, at defined intervals, periods of increased temperature and increased rainfall. Moreover, because temperature accelerates several steps in the process of mosquito and parasite development, the time lag between the appearance of suitable weather conditions and the appearance of new malaria cases should shorten as temperature rises. Although based largely on laboratory findings, these data suggest quantitative hypotheses about the precise time lag between increases in temperature and rainfall, and increases in malaria cases. At an average temperature of 20°C, the aquatic phase of the mosquito will be completed in about 28 days (five days for eggs to hatch and 23 days for the larva to develop into adult stage); and sporogony is completed in 28 days (Table 1). At this temperature, malaria cases should, therefore, appear 9–10 weeks following rainfall, assuming an average incubation period of about 10–16 days. Similarly, in this situation, the number of malaria cases should be positively related to increases in temperature three to seven weeks beforehand (during the aquatic and sporogony stages). When the mean temperature is higher (30°C), the aquatic stage of mosquito and the sporogony cycle are completed in about 12 and 8 days respectively (Table 1). In this relatively hot environment, malaria cases should appear 4–5 weeks following rainfall and the lag in the effect of temperature should also be shorter.

The purpose of the investigations reported here is to test these hypotheses using weekly data on weather and malaria cases. Specifically, we used Polynomial Distributed Lag Models (PDL) to determine the effect of weather factors and their lag distribution on malaria in relatively hot and cold environments using a data set consisting of weekly parasitologically confirmed malaria cases collected from health facilities and locally collected meteorological factors in 10 districts of Ethiopia for the years 1990 to 2000.

## Methods

### Data

Microscopically confirmed malaria cases were collected from a health facility in each of ten districts of Ethiopia over an average of 10 years. Each of these health facilities serve people living in the surrounding localities with few exceptions coming from other places. The data were extracted from records of outpatient consultations for the years 1990 through 2000. These data were compiled by residency (urban and rural) and *Plasmodium *species; the analysis was restricted to *P. falciparum*. The original data collected on the basis of Ethiopian weeks were normalized to obtain mean daily cases for each Ethiopian week [36].

Daily meteorological data (minimum and maximum temperature and rainfall) recorded at the local weather stations nearest to the health facility were obtained from the National Meteorological Services Agency (NMSA) for the same period. The assumption is that the meteorological data from the local weather stations represent the surrounding localities whose inhabitants are served by the respective health facilities. These daily data were collapsed into weekly data to correspond with the weekly malaria cases. The weekly mean for minimum and maximum temperature and the total weekly amount of rainfall were calculated from the daily records.

### Missing data

The data set consists of records with missing values for some of the variables (4.4%, 10.2% and 9.9% for rainfall, minimum and maximum temperature respectively). Because of the multiple time lags considered in the analysis (see below), discarding estimates with a single missing value results in multiple records lost for each missing value. To avoid this substantial loss of data, missing values of the independent variables (weather variables) were interpolated by fitting a linear regression using the value from the previous week and a dummy variable for that week. A new variable was created with original values for non-missing data points and interpolated values for missing data points.

### Model

The daily average number of cases (of the weekly microscopically confirmed malaria cases) was modeled using a robust Poisson regression (implemented in Splus), using weekly rainfall, minimum and maximum temperature as explanatory variables in a distributed lag model. The model was:



where *E*(*Y*_*st*_) denotes the expected value for the daily average number of malaria cases at site *s *on week *t*; ,  and *R*_*t - i *_are the weekly minimum and maximum temperature and the rainfall *i *weeks previously; *α*_*s *_and *β*_*s *_represent the intercept and coefficient for time trend (*t*), which are specific to the site under consideration.

As described in the introduction, biological considerations suggest that the lag between rainfall and associated malaria cases will be different from that between temperature and associated cases. Therefore, lags of 4–12 weeks were considered for rainfall, and relatively shorter lags of 3–10 weeks for the minimum and maximum temperature. The assumption is that changes in temperature and rainfall at a particular time do not have an important influence after 10 and 12 weeks respectively. The overall effect of a unit increase in minimum temperature (for example) is the sum of the coefficients *β*_*i*_.

A large number of variables (a total of 25; eight lags each for minimum and maximum temperatures and nine lags for rainfall) were introduced for the three weather factors in this model. The efficiency of the coefficient estimates may be affected due to the large number of parameters to be estimated and the possibility of multicollinearity between the lagged weather factors. When lag terms are put in the same model, correlation between measurements of weather on weeks close together will cause a high degree of collinearity that may result in unstable estimates.

### Polynomial Distributed Lag Model

A polynomial distributed lag (PDL) model [37] imposes constraints on the coefficients *β*_*i*_,*γ*_*i*_,*θ*_*i*_, forcing each of them to take the form of a (separate) 4^th^-degree polynomial in i. This reduces the number of degrees of freedom for each weather factor from the number of lags considered to five and circumvents some of the difficulties associated with estimation of coefficients for multiple lags, including instability of estimates due to collinearity of the different lags of the same variable. With this model the coefficients for lagged minimum temperature, for example, are assumed to take the form:



where *φ*_*k *_is the parameters of the d-th degree (here d = 4) polynomial distributed lag. To estimate the parameters describing the polynomial lag *φ*_*k *_equation (2) was substituted into the unconstrained distributed lag model (1) to obtain a constrained polynomial distributed lag model:



### Grouping of districts

The effects of weather factors on the number of malaria cases were distributed over multiple weeks and the separate analysis for each district (not shown) indicated heterogeneity in magnitude and direction of the effects of the weather factors. Districts with similar climatic characteristics were grouped, in order to reduce the effect of random error and to produce more reliable and precise estimates of weather effects. Moreover, this approach will produce more generalizable results within similar climatic conditions. Thus, the districts were grouped into hot (altitude < 1700 mm above sea level) and cold on the basis of altitude and temperature. The hot districts included Diredawa, Nazareth, Wolayita and Zeway; and the cold districts included Alaba, Awasa, Bahirdar, Debrezeit, Hosana and Jimma. Two separate PDL models were fitted to estimate the effects of different weather factors.

A basic issue in epidemiological analysis is controlling for the effect of confounding factors. Malaria transmission is affected by different factors and shows a systematic variation over time. To control for other factors that may affect the long-term trend, a time variable was included in the model, which would remove the long-term wavelength patterns, leaving the deviations representing short fluctuations. Since the long-term trend and the numbers of weekly cases vary between districts, an interaction term between the time variable and district (dummy variable) was introduced.

Urban areas may have other sources of breeding sites for mosquito not driven by rainfall. To examine the influence of these and other unmeasured factors that vary between urban and rural environments, separate models were fitted for urban and rural cases.

## Results

The data set consists of microscopically confirmed *P. falciparum *cases from a health facility in each of 10 districts over an average of 10 years and meteorological data from local stations in each of the districts. Table 2 presents the descriptive analysis of cases, meteorological variables and altitude of each district. The daily averages treated by each of the 10 health facilities ranged from 11–39 malaria cases and over 300 cases during the peak transmission season. Minimum temperature was positively correlated with rainfall, significantly (rho = 0.37) in the cold districts and nonsignificantly (rho = 0.06) in the hot. Maximum temperature, however, was negatively correlated with rainfall, significantly (rho = -0.33) in the cold districts and nonsignificantly (rho = -0.033) in the hot.

**Table 2:** Characteristics of the study districts (average daily malaria cases and meteorological variables)

District	Altitude	*Malaria cases*			Maxt	Mint	Rain	*Group*
	(m)	*Mean*	*Min*	Max	(°C)	(°C)	(mm)	
Alaba	1750	39	0	163	27.4	11.7	19.6	*cold*
Awasa	1750	11.3	0	17	27.4	12.6	19.3	*cold*
Bahirdar	1770	22.1	1	83	27.2	13	25.2	*cold*
Debrezeit	1900	25.2	1	147	26.5	12.1	16.5	*cold*
Hosana	2200	19.4	0	96	22.4	10.7	23.4	*cold*
Jima	1725	13.2	0	85	27.6	11.6	30	*cold*
Diredawa	1260	25.3	0	330	31.8	19	13.8	*hot*
Nazareth	1622	17.7	0	109	27.5	14.3	17.1	*hot*
Wolayita		13.9	0	113	25.3	14.4	24.4	*hot*
Zeway	1640	11.4	1	102	27.5	13.9	13.7	*hot*

maxt-maximum temperature, mint-minimum temperature

**Figure 1:**
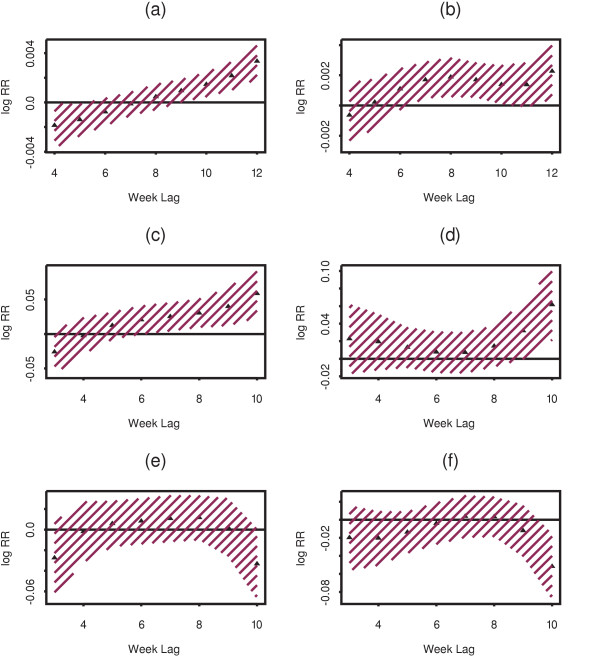
Distributed lag structure for the association between 1 mm increase in rainfall, 1°C increase in minimum and maximum temperature and average daily malaria cases. (a) & (b) for rainfall, (c) & (d) for minimum temperature, and (e) & (f) for maximum temperature in the cold and hot districts respectively. The shaded areas represent 95% confidence intervals.

The effect of rainfall and temperature on daily average microscopically confirmed cases was estimated by lag in the 10 districts grouped into two climatic zones, hot and cold (Table 2). Figure 1a shows the estimates of the distributed lag between rainfall and cases in cold areas. Coefficients represent the multiplicative effect of one additional millimeter of rain at a given lag on the number of malaria cases at a site. Rainfall is significantly associated with the number of malaria cases in the cold districts. The magnitude and direction of the association varies with lags. At shorter lags of 4 and 5 weeks, rainfall is negatively and significantly associated with malaria cases. At lags of six, seven and eight, rainfall is not significantly associated with malaria cases. Lags of nine, 10, 11 and 12 are positively associated with malaria cases and the magnitude of effect increases almost linearly with maximum effect at lag 12. The conclusion is that rainfall in the cold districts is associated with a much delayed increased malaria cases and immediate decrease in malaria cases.

Similarly, rainfall is significantly associated with malaria cases in the hot districts. Compared to the cold districts, a significant and positive effect of rainfall in the hot districts manifests at relatively shorter lags (six, seven, eight, nine and ten weeks) and remains positive afterwards but declines and becomes non-significant for the longer lags (Figure 1b). Much of the contribution of rainfall to the increase in malaria cases in the hot districts occurs at relatively shorter lags (compared to its effect in cold districts) and wanes slowly with increasing lags. Thus, the results for rainfall agree qualitatively with biological expectations.

Figure 1c shows the estimated distributed lag relationship between minimum temperature and malaria cases in the cold districts; coefficients represent the multiplicative effect of one degree Celsius increase in temperature at a given lag on the number of malaria cases at a site. Minimum temperature is positively associated with the number of malaria cases, with a significant increase extending from 7 to 10 weeks prior to cases and the size of the effect growing over that range.

In the hot districts, by contrast, the effect of minimum temperature on malaria cases is more complicated. At short lags, its effect is small and non-significantly positive (Figure 1d). A significant positive association at longer lags is also observed. In summary, minimum temperature contributed significantly to the estimated increase in malaria cases in the cold districts with a delayed effect. In the hot districts, while its effect is non-significant, much of the contribution is relatively immediate. Unexpectedly, the only significant contribution of minimum temperature in the hotter districts occurs at long lags.

Figures 1e and 1f show the relationship between maximum temperature and malaria cases in the cold and hot district groups respectively. Maximum temperature is not significantly associated with the estimate of malaria cases in either group of districts. However, the trend of the estimates along the lags shows that at shorter lags of three, four and five weeks, maximum temperature is negatively associated with the number of malaria cases in hot districts, while it is positively associated at lags of six, seven and eight weeks in the cold districts.

To test the linearity of the association between the weather factors and malaria cases, a three dimensional relationship between weather factors, lag and the magnitude of effect at each lag was explored (See Additional file 1 for the method and figure). In that figure, a positive effect of a factor at a given lag is seen as a positive slope of the surface cut at the given lag; the magnitude of that slope corresponds to the linear effect estimated by the PDL model. The effect of rainfall plateaus at higher rainfall levels; beyond a given quantity of rain, additional rain adds little to the malaria risk. Similarly, although the effect of minimum temperature in the cold districts is linear, it levels off at higher temperature (>16°C) in the hot districts.

**Figure 2:**
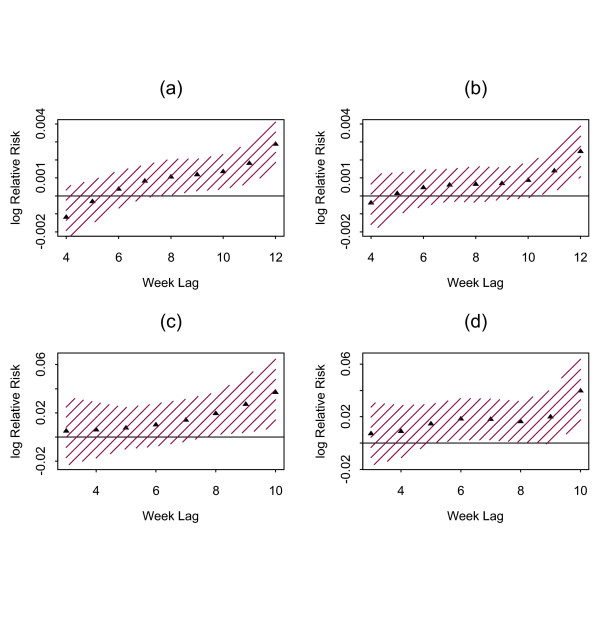
Distributed lag structure of the effects of rainfall and minimum temperature on average daily malaria cases by residency. (a) & (b) for rainfall in rural and urban respectively, (c) & (d) for minimum temperature in rural and urban respectively. The shaded areas represent 95% confidence intervals.

The results of analysis stratified by rural versus urban sites are shown in Figure 2. The association between rainfall and cases varies by residency (a & b). Rainfall is significantly associated with cases originated from rural residents but not generally among urban residents, however, the magnitude of effect of rainfall looks similar in both rural and urban areas,. The effect of minimum temperature on malaria cases does not vary by residency (c & d).

**Figure 3:**
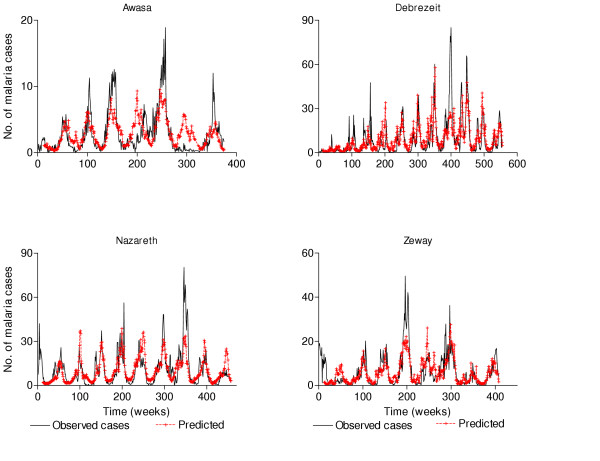
Plot of observed number of cases and predicted cases from the polynomial distributed lag models of 4 districts.

Predicted case numbers for hot and cold districts were compared against actual values to assess how well the models predict the seasonal peaks and interannual variability. Plots of the actual data and predicted values showed that the models predicted the seasonal fluctuations very well (Figure 3). However, the models were not able to differentiate clearly between years with high and low peaks.

## Discussion

The development of malaria early warning systems [7,8] to predict where and when malaria epidemics will occur, in order to target scarce resources for prevention activities [9,38], has motivated many studies [13-15,18,19]. However, little consensus has emerged as to which factors should be used as indicators, because multiple studies have yielded differing results on the main determinants of increased malaria transmission [2,6,21-23,25,27] and the lead time prior to observable effects [3,4,22,24,29]. In this study a polynomial distributed lag model was used to assess the lag distribution of the effects of weather factors on Plasmodium *falciparum *malaria in relatively hot (Diredawa, Nazareth, Wolayita and Zeway) and cold (Alaba, Awasa, Bahirdar, Debrezeit, Hosana and Jimma) environments in Ethiopia.

The findings are largely consistent with hypotheses based on the relationship between weather factors and mosquito and parasite development. Rainfall is associated with malaria cases in both hot and cold districts with a lagged effect, and as expected, this lag is shorter in hot districts. The effect of rainfall on malaria is linear with saturating effects at higher rainfall levels (See Additional file 1 for the method and figure). Interestingly, malaria in the urban areas is not associated with rainfall. Although the maximum temperature is not generally associated with malaria cases in either group of districts, the minimum temperature is significantly associated with malaria cases in the cold districts with delayed effect, and the lag for the minimum temperature is shorter than that for rainfall, reflecting the two factors' effects on different stages of the transmission cycle. The detection of a positive effect of the minimum temperature at long lags (9–10 weeks) in the warmer districts was not predicted by biological considerations.

One of the most striking uncertainties in the literature on weather and malaria is the variability in the reported relationship between rainfall and malaria, with several studies showing the importance of rainfall as a precipitating factor for malaria transmission [3,4,10,11,29], while other studies show negative or neutral effects [21,23,26,27]. For rainfall to have a positive effect on malaria cases, the temperature must be warm enough to support mosquito and parasite development [39], and, as the data confirm, the effect of rainfall on cases becomes more immediate in warmer temperatures. This is consistent with the laboratory findings that a mosquito population peaks early at higher temperatures, while a mosquito population at low temperatures experiences slow, steady growth with a delayed peak [40]. Increases in rainfall may also fail to produce additional malaria cases if aquatic breeding sites are not limiting for mosquitoes; this mechanism is consistent with the observed saturating effect of rainfall in our data.

Furthermore, malaria in the urban areas is not significantly associated with rainfall, which may have been one of the sources of inconsistent findings of such analysis. The weak association may be due to the presence of other sources of breeding sites that may persist during the dry season such as brick pits, puddles, blocked drains and cisterns [41]. Moreover, developmental activities, aggregation of migrant labor forces and overall population movement affect urban malaria. It is also interesting to note that the effect of rainfall in the cold districts is negative at shorter lags, which may be due to breeding sites being flushed away during the rainy season [23]. Another possible explanation for the negative effect could be that low temperature during the rainy season might suppress malaria transmission. Maximum temperature was lower during the rainy season (shown by negative correlation with rainfall), however, the effect of maximum temperature on malaria is non significant. Moreover, minimum temperature seems to be elevated during the rainy season (positive correlation). Although the effect of rainfall in the hot districts declines after longer lags (due to evaporation and drying up of breeding sites), making the main transmission season shorter, the overall effect of rainfall (sum of the lag coefficients) is bigger in the hot than cold districts. Taken together, the analyses suggest that temperature requirements, saturating effects of rainfall, and urban-rural differences in the effect of rain on malaria transmission are all plausible mechanisms that could explain the inconsistent relationship between excessive rainfall and malaria epidemics.

The minimum temperature contributes significantly to the estimated increase in malaria cases with a delayed effect in the cold districts, but not in the hot districts. At lower temperatures, the larval and pupal stages of mosquitoes take longer to complete (for example, 47 days at 16°C) and a small increase in temperature substantially shortens the duration of these phases (to 37 days at 17°C). Similarly, the duration of the sporogony cycle will be short with increasing temperatures (Table 1). In addition, raised temperature increases the frequency of mosquito feeding and, hence, the probability of transmitting infection [33]. Although all such effects of minimum temperature increase malaria transmission in the cold districts, the effect will be seen after a lag. The effect of minimum temperature in the hot districts, on the other hand, is immediate but non-significant. These findings are consistent with reports that small increases in temperature will have a greater effect on malaria transmission in areas with relatively lower average temperatures than areas with higher temperatures [42,43]. The significant effect of minimum temperature at relatively long (9–10 week) lags is not explainable, to our knowledge, on biological grounds.

The maximum temperature is not significantly associated with cases in either hot or cold districts. However, the negative (but non-significant) correlation between weekly malaria cases and maximum temperature at shorter lags seen in hot districts may be due to its inhibitory and lethal effect on the survival of the parasites in the mosquitoes [30]. The survival rate of *Anopheles gambiae *is also reduced at higher temperatures. Nonetheless, the maximum temperature is not very extreme even in the relatively hot districts, thus the negative effect is not significant.

As with all observational studies of malaria incidence and weather, a limitation of this study is the likely presence of some confounding factors that may have influenced the number of malaria cases and may have been associated with weather. Existing interventions such as insecticide residual spraying and other methods are routinely applied and were not included in this analysis. The results would have been biased by such confounding factors if interventions were undertaken on the basis of weather, or if they were undertaken on the basis of incidence and their effect was differential depending on the weather. Another minor problem is with the assumption of a finite length for the delayed effect of the different weather factors. However, the lag length was chosen based on the inter-relationship between weather, mosquitoes and parasites (Table 1) and it was assumed that this is biologically plausible.

Weather factors alone explain seasonal cycles but were not accurate in explaining the magnitude of unusually bad years (Figure 3). This study was a scientific not a predictive exercise and suggests that no other factor is required for explaining seasonal cycles. A good early warning system has not been created, but some principles have been suggested for one. The first principle is that the lag length from time of rain to the expectation of malaria cases varies with climatic zone (with a saturating effect at higher rainfall levels), and rainfall may not be a key factor in urban malaria transmission. Secondly, minimum temperature is only important in the cold climatic zones, but not in the hot. Finally, maximum temperature makes little difference in either climatic zone. These key points need to be considered in the development of an early warning system for malaria. Such an early warning system would also include autoregressive terms or other terms that could improve prediction, but would have complicated the interpretation of coefficients in a model of the sort used, which was designed to detect the effects of weather factors on cases, rather than to predict case numbers. Such an early warning system is now being evaluated.

## Conclusions

The findings are largely consistent with hypotheses, based on experimental data on mosquito and parasite development, about the interactions of climatic factors in determining the strength and lag structure of weather effects on falciparum malaria incidence. In the examined Ethiopian districts, weather-based predictors of malaria incidence are more useful in rural than in urban settings. These key points should be considered in the development of an early warning system for malaria.

## Authors' contributions

HDT and ML conceived the study, undertook statistical analysis and drafted the manuscript. AT initiated the study and made data available in collaboration with WHO and Ministry of Health of Ethiopia. JS made major contributions to the study design and statistical analysis. All authors contributed to the writing of the manuscript and approved the submitted version of the manuscript.

## Supplementary Material

Additional File 1 Three dimensional relationships between weather, lags and magnitude of effect at each lag Click here for file
