# Supplementary material for: Weather-based prediction of Plasmodium falciparum malaria in epidemic-prone regions of Ethiopia I. Patterns of lagged weather effects reflect biological mechanisms
Source: Malar J. 2004 Nov 12;3:41. doi: 10.1186/1475-2875-3-41 (PMC535540; doi:10.1186/1475-2875-3-41)
Supplement: Additional File 1 — Three dimensional relationships between weather, lags and magnitude of effect at each lag [file 1475-2875-3-41-S1.pdf]

Three dimensional relationships between weather, lags and magnitude of effect at each lag

The figures presented in the main text are two dimensional figures with the weekly lags as the x-axis and the log of relative risk as the y-axis. To test the log-linearity of the association between the weather factors and malaria cases, we explored a three dimensional relationship between weather factors, lags and magnitude of effects at each lag. For this purpose, we fitted a polynomial distributed lag model with linear and quadratic terms for each factor in the model, and quartic lag structure as in the main analysis.

$$Log(E(Y_{st})) = \alpha_s + \sum_{i=3}^{10}(\sum_{k=0}^{4}\phi_k i^k)T_{t-i}^{(\min)} + \sum_{i=3}^{10}(\sum_{k=0}^{4}\phi^*{}_k i^k)\,sqT_{t-i}^{(\min)} + \sum_{i=3}^{10}(\sum_{k=0}^{4}\chi_k i^k)T_{t-i}^{(\max)} + \sum_{i=3}^{10}(\sum_{k=0}^{4}\chi^*{}_k i^k)sqT_{t-i}^{(\max)} + \sum_{i=4}^{12}(\sum_{k=0}^{4}\psi_k i^k)R_{t-i} + \sum_{i=4}^{12}(\sum_{k=0}^{4}\psi^*{}_k i^k)sqR_{t-i} + \beta_s t$$

where $E(Y_{st})$ denotes expected value for the weekly number of malaria cases at site *s* on week *t*, $T_{t-i}^{(\min)}$, $T_{t-i}^{(man)}$ and R_{t-i} are the weekly minimum and maximum temperature, and rainfall; and $sqT_{t-i}^{(\min)}$, $sqT_{t-i}^{(man)}$ and sqR_{t-i} are the square of the weekly minimum and maximum temperature, and rainfall i weeks previously. ϕ_k, χ_k, ψ_k and $\phi^*{}_k, \chi^*{}_k, \psi^*{}_k$ represent the parameters of the k-th degree polynomial distributed lag for the linear and quadratic terms of minimum and maximum temperature and rainfall respectively. γ_0 represents the intercept. In addition district was recoded to create dummy variables (location). An interaction term of location and time was created and included in the model.

To create the three dimensional plots, the coefficients for the effects of the weather factors at each lag were estimated and stored in a matrix, and plotted as a series of joined curves against the value of each weather factor. Supporting Figure 1 shows the relationship between the logarithm of the number of weekly malaria cases and each of the weather factors considered in the analysis at each lag. A positive effect of a factor at a given lag is seen as a positive slope of the surface cut at the given lag; the magnitude of that slope corresponds to the linear effect estimated by the PDL model. The effect of rainfall is linear at smaller amount of rainfall levels and then flattens out at higher levels of rainfall (approximately > 90 mm per week). The slope for the effect of minimum temperature in the cold districts is linear. While the trend of the effect of minimum temperature in the hot districts is linear up to temperature of 16°C, it levels off at higher temperature. Similar to the 2-dimensional plots, the 3-dimensional plots for maximum temperature also show no association. The slope of each line is not different from zero i.e. there is no trend and each line is parallel to the axis with temperature measurements.

Supporting Figure 1: Distributed lag structure for the association between an increase in rainfall, in minimum and maximum temperature, and average daily malaria cases. (a) & (b) for the effect of rainfall, (c) & (d) for the effect of minimum temperature, and (e) & (f) for the effect of maximum temperature in the cold and hot districts respectively.

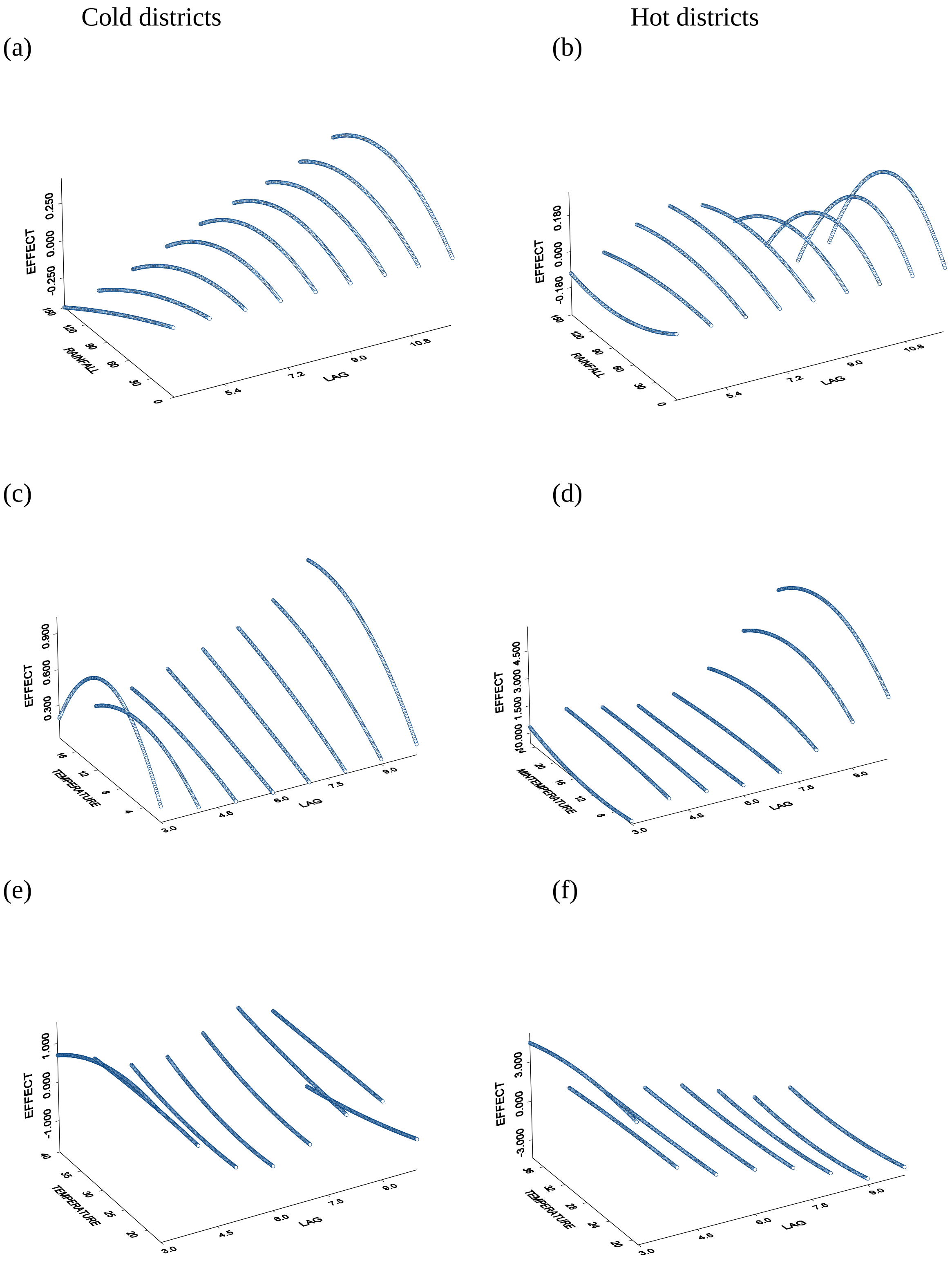
Cold districts
Hot districts
(a)
(b)
EFFECT
RAINFALL
LAG
(c)
(d)
EFFECT
TEMPERATURE
MINTEMPERATURE
LAG
(e)
(f)
EFFECT
TEMPERATURE
LAG
